# Intraspecific plant–soil feedback and intraspecific overyielding in *Arabidopsis thaliana*

**DOI:** 10.1002/ece3.1077

**Published:** 2014-05-24

**Authors:** Alexandra R Bukowski, Jana S Petermann

**Affiliations:** 1 Freie Universität Berlin, Institute of BiologyKönigin-Luise-Straße 1-3, Berlin, 14195, Germany; 2 Berlin-Brandenburg Institute of Advanced Biodiversity Research (BBIB)Altensteinstr. 6, Berlin, 14195, Germany

**Keywords:** *Arabidopsis thaliana* accessions, community ecology, diversity–productivity relationship, ecosystem functioning, home-away effect, intraspecific diversity, plant coexistence, plant–soil (below-ground) interactions, trait measurements

## Abstract

Understanding the mechanisms of community coexistence and ecosystem functioning may help to counteract the current biodiversity loss and its potentially harmful consequences. In recent years, plant–soil feedback that can, for example, be caused by below-ground microorganisms has been suggested to play a role in maintaining plant coexistence and to be a potential driver of the positive relationship between plant diversity and ecosystem functioning. Most of the studies addressing these topics have focused on the species level. However, in addition to interspecific interactions, intraspecific interactions might be important for the structure of natural communities. Here, we examine intraspecific coexistence and intraspecific diversity effects using 10 natural accessions of the model species *Arabidopsis thaliana* (L.) Heynh. We assessed morphological intraspecific diversity by measuring several above- and below-ground traits. We performed a plant–soil feedback experiment that was based on these trait differences between the accessions in order to determine whether *A. thaliana* experiences feedback at intraspecific level as a result of trait differences. We also experimentally tested the diversity–productivity relationship at intraspecific level. We found strong differences in above- and below-ground traits between the *A. thaliana* accessions. Overall, plant–soil feedback occurred at intraspecific level. However, accessions differed in the direction and strength of this feedback: Some accessions grew better on their own soils, some on soils from other accessions. Furthermore, we found positive diversity effects within *A. thaliana*: Accession mixtures produced a higher total above-ground biomass than accession monocultures. Differences between accessions in their feedback response could not be explained by morphological traits. Therefore, we suggest that they might have been caused by accession-specific accumulated soil communities, by root exudates, or by accession-specific resource use based on genetic differences that are not expressed in morphological traits. *Synthesis*. Our results provide some of the first evidence for intraspecific plant–soil feedback and intraspecific overyielding. These findings may have wider implications for the maintenance of variation within species and the importance of this variation for ecosystem functioning. Our results highlight the need for an increased focus on intraspecific processes in plant diversity research to fully understand the mechanisms of coexistence and ecosystem functioning.

## Introduction

The loss of biodiversity is a major global problem that is currently being accelerated by climate change and other man-made stressors such as overexploitation and pollution (Millennium Ecosystem Assessment 2005). Plants as sessile organisms are under strong pressure to adapt to changing conditions in order to escape extinction. These changes do not only affect individuals or species but also their interactions with other organisms (Wardle et al. 2011). For example, individual plants may be affected by conspecifics, heterospecifics as well as above- and below-ground herbivores and pathogens. However, we still have limited knowledge of the major mechanisms and consequences of these interactions for the coexistence of species in diverse natural communities and for ecosystem functioning.

Investigating the effects of plant species on their associated soil communities and *vice* versa via plant–soil feedback, one may distinguish between positive and negative feedback (Bever et al. 1997; Bever et al. 2012; Bever 2003; van der Putten et al. 2013). In general, positive plant–soil feedback can lead to the dominance of certain plant species at a site, therefore causing a loss of biodiversity. It has been invoked to explain the success of invasive plant species (Klironomos 2002; Reinhart et al. 2003; Callaway et al. 2004; Reinhart and Callaway 2004; Agrawal et al. 2005). In contrast, negative plant–soil feedback is thought to contribute to the maintenance of species diversity (Bever et al. 1997). It is essentially a Janzen–Connell-type mechanism that generates negative density dependence (Janzen 1970; Connell 1971). Thus, it operates as stabilizing effect with the potential to maintain plant coexistence (Chesson 2000). Studies have shown that negative feedback is more common than positive feedback (Kulmatiski et al. 2008); however, there might be a bias in detecting negative effects (Bardgett and Wardle 2010).

Furthermore, plant–soil feedback may play a key role in the positive diversity–productivity relationship that has been found across plant communities (Schnitzer et al. 2010; Maron et al. 2011; Kulmatiski et al. 2012; Hendriks et al. 2013). This relationship is also referred to as “overyielding,” that is, plants growing in species mixtures produce more biomass than plants growing in species monocultures (Loreau and Hector 2001; Tilman et al. 2001; Hector et al. 2002; Schnitzer et al. 2010; Maron et al. 2011; Kulmatiski et al. 2012; Hendriks et al. 2013). The positive diversity effect had traditionally been attributed to resource-niche complementarity (Tilman et al. 1996; Fargione and Tilman 2005). However, “pathogen niches” (the collective effects of pathogens specific to each host plant species, see Petermann et al. (2008)) emerging from plant–soil feedback may also contribute to the positive diversity–productivity relationship (Westover and Bever 2001).

**Figure 1:**
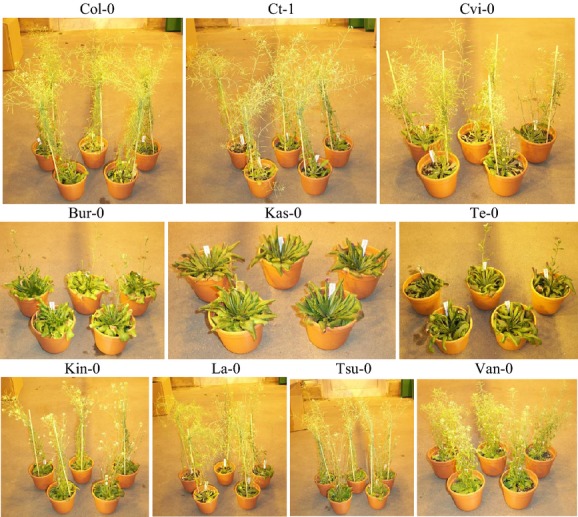
*Arabidopsis thaliana* accessions that were chosen for the plant–soil feedback experiment. The pictures show 7-week-old plants (five individuals of each accession) on which the above-ground trait measurements were taken (photographs by Alexandra R. Bukowski).

So far, little is known about the exact mechanisms of soil communities driving above-ground dynamics of plant communities. However, there is evidence that plant species, even if they are closely related, accumulate distinct bacterial and fungi soil communities and that plant performance varies when growing in soils differing in the composition of soil communities (Pendergast et al. 2013). Generally, intraspecific diversity in plants has recently been suggested to be important for ecological processes (Albert et al. 2011; Bolnick et al. 2011). With few exceptions (Smith et al. 2012), virtually all experiments examining plant–soil feedback have been performed at species level comparing the growth of one species on home soil (species A on soil from species A) versus away soil (species A on soil from species B). In contrast, the aim of our study was to investigate whether plant–soil feedback operates at intraspecific (interaccession) level, analogously comparing the growth of plants of accession A on soil from accession A (home soil) versus soil from other accessions (away soil). We tested this using 10 available natural accessions of the model species *Arabidopsis thaliana* (L.) Heynh. (Fig. 1). To our knowledge, there is only one study that conducted feedback experiments in *A. thaliana*. Aguilera et al. (2011) used different *A. thaliana* accessions originating from different locations around the world and found that plants grew better in sterile soil than in soil “trained” (previously occupied) by certain accessions. Furthermore, the composition of the background community affected the growth behavior of the focal plant, that is, some accessions had either stronger or weaker effects on the focal plants than other accessions. The authors speculated that their results might be linked to potential morphological differences between the accessions. Other experiments have shown that *A. thaliana* accessions differ in their root morphology (Scheres et al. 2002; Mouchel et al. 2004; Shindo et al. 2008; Pacheco-Villalobos and Hardtke 2012). Moreover, *A. thaliana* accessions have been found to differ in the release of root exudates as well as in the accumulation and composition of rhizobacterial communities (Micallef et al. 2009; Bulgarelli et al. 2012; Lundberg et al. 2012). *Arabidopsis thaliana* seedlings have also been shown to develop differences in root morphology when coming in contact with root exudates of their own versus another accession (Biedrzycki et al. 2010). Because *A. thaliana* constitutes one of the exceptions in the plant world by not being colonized with arbuscular mycorrhizal fungi, it is especially suitable for studies examining its root microbiota. For example, a recent study revealed that the effects of fungal endophytes differ depending on the identity of the host accession as well as on the fungal strain (Mandyam et al. 2013). Thus, dynamics between plants and their associated soil communities might be determined partly at intraspecific level. Apart from differences in root morphology, *A. thaliana* accessions, as well as more generally wild types and mutants, show differences in above-ground traits (Li et al. 1998; Frenkel et al. 2007; Passardi et al. 2007). This variation among *A. thaliana* accessions may be a result of adaptation and selection processes. After its first appearance five million years ago, *A. thaliana* spread all over the world and populations adapted to different environmental conditions whenever reaching a new climatic zone (Alonso-Blanco and Koornneef 2000; Koch et al. 2000). Experiments have indeed shown that there is a negative correlation between latitude and plant performance in many *A. thaliana* accessions, that is, the higher the latitude of the origin, the smaller the relative growth rate and parameters of plant size (Li et al. 1998).

We conducted this study to test whether *A. thaliana* experiences soil feedback at intraspecific level, whether different accessions vary in the strength of this feedback, and whether differences might be linked to morphological traits. We also examined whether the plants' above-ground biomass differs when growing in monoculture (intra-accession competition) or in mixture (interaccession competition) and whether a difference in the individual performance increases ecosystem function at mixture level. We hypothesized that (1) the *A. thaliana* accessions differ in their above-ground as well as below-ground traits, (2) plant–soil feedback operates at intraspecific level and that the *A. thaliana* accessions differ in the strength of the feedback they experience possibly due to different morphological traits, (3) *A. thaliana* shows positive intraspecific diversity effects (intraspecific overyielding), more strongly so in trained compared with neutral soil.

## Material and Methods

### Experimental species and plant material

*Arabidopsis thaliana* (Brassicaceae) is a relatively small annual flowering and self-pollinating plant species that completes its entire life cycle in 6–8 weeks and has an r-reproductive strategy (i.e., one individual is able to produce thousands of seeds).

We chose 10 *Arabidopsis thaliana* accessions from a number of locations around the world in order to include as much natural variation as possible (Fig. 1, Table 1). Criteria for the choice of those accessions were a priori information on differences in their morphology, especially in root traits where information was available (Mouchel et al. 2004; Passardi et al. 2007; Shindo et al. 2008; Micallef et al. 2009; Aguilera et al. 2011; Pacheco-Villalobos and Hardtke 2012). We used seeds provided by the Nottingham Arabidopsis Stock Centre (NASC) and by several research groups of the Freie Universität Berlin being associated with the Dahlem Centre of Plant Sciences (DCPS).

**Table 1:** List of the 10 *Arabidopsis thaliana* accessions and their origins

Accession	Origin	Latitude
Bur-0	Burren, Ireland	53.1486°N
Col-0	Columbia, MO	38.9517°N
Ct-1	Catania, Italy	37.5080°N
Cvi-0	Cape Verde Islands	16.0000°N
Kas-1	Kashmir, India	34.1491°N
Kin-0	Kendalville, MI	41.4414°N
La-0	Landsberg, Poland	52.7325°N
Te-0	Tenala, Finland	60.0585°N
Tsu-0	Tsu, Japan	34.7186°N
Van-0	Vancouver, Canada	49.2612°N

### Growing conditions and soil composition

All studies described here were conducted between November 2012 and March 2013. Plants were grown in a greenhouse at Freie Universität Berlin at a humidity of 60% and under long-day conditions, that is, 16-h light (day temperature: 25°C) and eight-hour darkness (night temperature: 20°C). Light intensity was 120 *μ*mol quanta/m^2^/s supplemented by high-pressure sodium lamps (2000K; Philips Powertone Son-T Agro, Hamburg, Germany). For all studies and experiments, we used premixed soil (Einheitserde- und Humuswerke Gebr. Patzer GmbH & Co. KG [Sinntal-Altengronau, Germany], composition: 50% organic substances, 1.7 g/L KCl, 194.5 mg/L CaCl_2_, 189 mg/L P_2_O_5_, 267 mg/L K_2_0, pH = 5.8) consisting of white peat, clay, and perligran G (Knauf Aquapanel GmbH, Dortmund, Germany).

### Trait measurements

We measured a number of above- and below-ground traits in the chosen *A. thaliana* accessions in order to assess the degree of intraspecific variation between these accessions (Table 3). Based on these results, we designed the experiment phase of the plant–soil feedback experiment (see below and Table 2) by matching each accession (1) with one similar accession (for the “away similar” soil type) and (2) with one dissimilar accession (for the “away different” soil type).

**Table 2:** Assignment of soil types for the monocultures of the experiment phase according to the results of the above- and below-ground trait measurements. Each of the 10 soil types belonging to the 10 accessions was used exactly once as “home soil” (trained by an accession being the same as the accession of the monoculture), once as “away similar soil” (trained by an accession assessed as similar to the accession of the monoculture) and once as “away different soil” (trained by an accession assessed as dissimilar to the accession of the monoculture)

Monoculture	Home soil	Away similar soil	Away different soil
Bur-0	Bur-0	Kas-1	Ct-1
Col-0	Col-0	Ct-1	Te-0
Ct-1	Ct-1	La-0	Cvi-0
Cvi-0	Cvi-0	Kin-0	Col-0
Kas-1	Kas-1	Te-0	Kin-0
Kin-0	Kin-0	Tsu-0	Kas-1
La-0	La-0	Van-0	Bur-0
Te-0	Te-0	Bur-0	La-0
Tsu-0	Tsu-0	Col-0	Van-0
Van-0	Van-0	Cvi-0	Tsu-0

Mean dissimilarity coefficients ± SE: 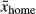 = 0 ± 0, 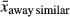 = 0.21 ± 0.02, 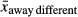 = 0.43 ± 0.05.

#### Above-ground trait measurements

After having been stratified in dry condition at 4°C for 5 days, seeds were sown in pots (height 10 cm, diameter 11 cm) filled with autoclaved soil. For this study, each plant was grown individually in one pot. To ensure the presence of one germinated seedling in each pot, three seeds were sown and additional germinated seedlings were removed after germination. In total, there were 50 pots (10 accessions × 5 replicates). After sowing, pots were watered from above (sprayed) and below (individual trays with water). Plants were watered daily in the first 2 weeks and four to five times a week for the remainder of the study. The position of the pots was rerandomized once to twice a week. When the siliques ripened, we counted the number of seeds in three randomly chosen mature siliques of each plant to calculate the average number of seeds per silique and estimate the average number of seeds per plant. We measured stem height, rosette diameter as well as number of siliques of each plant and harvested them 7 weeks after sowing. Then, we dried the plants for 4 days at 60°C to determine the above-ground biomass (dry weight).

#### Below-ground trait measurements

For this study, we used 10-day-old *A. thaliana* seedlings to be able to extract the root system from the soil. For each of the 10 accessions, 15 seeds were sown in smaller pots (height 6 cm, diameter 9 cm) filled with autoclaved soil. Until the harvest, all pots were sprayed and rerandomized almost daily. Ten days after germination, five seedlings of each accession were harvested. Each of those seedlings was extracted from the pot as a whole taking care not to damage the roots. After cutting the shoots, the entire root systems were washed and stored in water-filled glass tubes. These root samples were scanned and analyzed by the WinRHIZO (Regent Instruments Inc., Sainte-Foy, Quebec City, Canada). We focused on four important traits: root length, root surface area, root volume, and average root diameter.

### Plant–soil feedback experiment

Following the approach common to most plant–soil feedback experiments (Kulmatiski et al. 2008, 2012; Petermann et al. 2008; Aguilera et al. 2011; van de Voorde et al. 2011; Reinhart 2012; Hendriks et al. 2013; Pendergast et al. 2013), we conducted two phases. For the first phase (training phase), we used autoclaved soil. *Arabidopsis thaliana,* as an annual plant species, which are often dominant on anthropogenic soils during early succession (Rebele 1992), was expected to rapidly accumulate an own suite of microorganisms. During the second phase (experiment phase), we tested whether this soil training had an effect on a new generation of plants. For both phases, general methods followed the same protocol as used for the above-ground trait measurements. In all plants of the plant–soil feedback experiment, we measured above-ground biomass, stem height, rosette diameter, and number of siliques.

#### Phase 1: training phase

The training phase consisted of two training types: monoculture training and mixture training. Each pot contained 10 plants that were used to train the soil. For the monoculture training, seeds of one accession were placed in a circle of 10 in a pot. This setup was replicated five times for each of the 10 accessions. For the mixture training, seeds of all 10 accessions were sown together in a pot, with a total of 10 seed locations (one location per accession) arranged in a circle. Seed locations were marked so that accessions could be identified later on. This setup was replicated 10 times. Overall, there were 60 pots (10 monocultures × 5 replicates + 1 mixture × 10 replicates) with a total of 600 plants for the training phase. When the first siliques ripened (six and a half weeks after sowing), all plants were harvested in order to prevent seeds from dropping into the soil and influencing the experiment phase. The soil was stored for 2 weeks at cold temperatures until the start of the experiment phase.

#### Phase 2: experiment phase

One day before sowing, the soil was prepared by homogenizing in order to distribute the roots of the training plants as evenly as possible. All replicates of a soil type were mixed with an equal volume of autoclaved soil to dilute abiotic effects. During the experiment phase, plants were growing again both in monoculture as well as in mixture. However, each monoculture was grown in three different soil types: (1) on “home soil” (trained by the same accession), (2) on “away similar soil” (trained by an accession assessed as similar according to the results of the trait measurements, see above and Table 2), and (3) on “away different soil” (trained by an accession assessed as dissimilar according to the results of the trait measurements, see above and Table 2). This setup was replicated three times for each monoculture and each soil type. In contrast to that, the mixtures only grew in one soil type, that is, on “mixture-trained soil.” This was replicated five times. In total, there were 95 pots (10 monocultures × 3 soil types × 3 replicates + 1 mixture × 1 soil type × 5 replicates) with a total of 950 plants for the experiment phase.

### Statistical analyses

For all analyses, we used the software R version 3.0.0 (R Development Core Team 2013). To analyze the differences between the *A. thaliana* accessions in the measured above-ground and below-ground traits, we used multivariate analysis of variance (MANOVA). Furthermore, we created a dissimilarity matrix based on Gower (1971) dissimilarity coefficients [R package “FD”, function gowdis() (Laliberté and Shipley 2011)]. In this calculation, we included all measured traits in order to select pairs of accessions that were most similar and dissimilar.

Following the common approach of calculating the feedback strength, we primarily focused on above-ground biomass of the plants of the experiment phase (Petermann et al. 2008; van de Voorde et al. 2011; Kulmatiski et al. 2012; Reinhart 2012; Pendergast et al. 2013). We calculated the soil feedback that each accession experienced on “home soil” versus “away soil” as a logarithm-transformed ratio of the above-ground biomass following Petermann et al. (2008). Plant pairings for the ratios were randomized, that is, data from the 30 plants growing on “home soil” (three pots with 10 individuals each) were randomly combined with (1) the data from the 30 plants growing on “away similar soil” and with (2) the data from the 30 plants growing on “away different soil,” respectively. This resulted in 60 values per accession. As there were no significant differences between “away similar” and “away different,” we decided to use the average of the 60 values. To test whether the 10 accessions differed in the soil feedback they experienced, we used those calculated values as a response variable in mixed-effects models with “pot” as random effect because measurements at individual plants within a pot cannot be considered independent [R package “nlme”, function lme() (Pinheiro et al. 2013)].

In order to explain the variance between accessions, we calculated a number of indices and tested them as explanatory variables in mixed effect models with “accession” as random effect. Importantly, the calculation of these indices was based on measurements from another set of plants than the feedback calculation. Therefore, we related accession-level averages of traits and indices (from the trait measurement and training phase) to soil feedback effects (measured in the experiment phase). The indices were as follows:

- average above-ground biomass, root length, root surface area, root volume, or root diameter per individual as determined by the trait measurements (5 plants per accession);
- average above-ground biomass in monocultures during the training phase (50 plants per accession);
- average above-ground biomass in mixtures during the training phase (10 plants per accession);
- relative above-ground biomass in mixtures during the training phase (the accession's share of the total above-ground biomass per pot, 10 plants per accession);
- the latitudes of the accessions' origins.

To test whether the total above-ground biomass per pot differed between the two phases (training phase vs. experiment phase) and between the two community types (monocultures vs. mixtures), we used linear models.

## Results

### Above- and below-ground trait measurements

**Figure 2:**
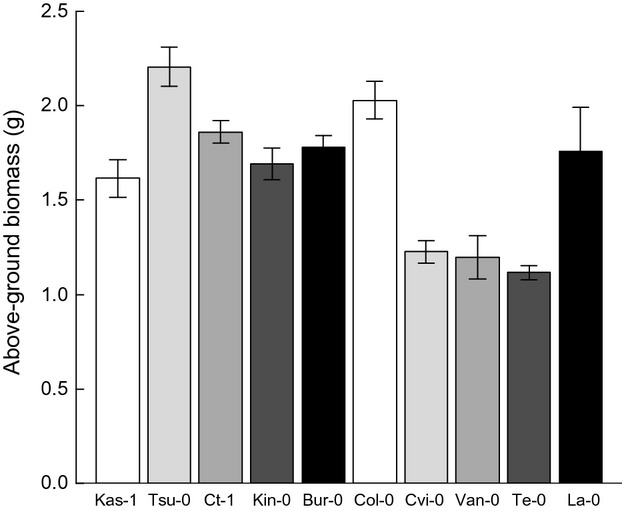
Variation in the above-ground biomass per individual in g of the 10 *Arabidopsis thaliana* accessions determined in the above-ground trait measurements. The order of accessions in the graph corresponds to increasing feedback strength (see Fig. 3, colors facilitate the comparison). Bars represent the mean ± standard error. For the statistical analysis, see Table S1. *n* = 5 for each accession.

The 10 *Arabidopsis thaliana* accessions differed significantly in their above-ground traits (above-ground biomass (Fig. 2), stem height, rosette diameter, number of siliques, average number of seeds per silique, average number of seeds per plant, see Tables 3 and S1) as well as in their below-ground traits (root length, root surface area, root volume, average root diameter, see Tables 3 and S1). The above-ground biomass of the smallest accessions Cvi-0, Te-0, and Van-0 was about 50% smaller than the biomass of Col-0 and Tsu-0 (Fig. 2, Table 3). The stem height of the accessions Te-0 and La-0 differed approximately by a factor of four (Table 3). Kas-1 was the only accession that did not grow stems. The root volume of Van-0 was about twice as large as of Ct-1 (Table 3).

**Table 3:** Mean trait values of the 10 *Arabidopsis thaliana* accessions determined in the trait measurement study as well as in the plant–soil feedback experiment. Values represent the mean ± SE. For the statistical analysis, see Tables S1 and S3. The number of replicates (*n*) is given in brackets

	Bur-0	Col-0	Ct-1	Cvi-0	Kas-1	Kin-0	La-0	Te-0	Tsu-0	Van-0
**Above-ground trait measurements (*n* = 5)**										
Above-ground biomass (g)	1.78 ± 0.06	2.03 ± 0.09	1.86 ± 0.05	1.23 ± 0.05	1.61 ± 0.09	1.69 ± 0.08	1.76 ± 0.21	1.12 ± 0.03	2.21 ± 0.09	1.20 ± 0.10
Stem height (cm)	24.30 ± 3.90	55.10 ± 0.99	61.40 ± 0.46	38.80 ± 1.40	0.00 ± 0.00	46.00 ± 2.54	65.50 ± 1.82	15.00 ± 4.03	55.10 ± 1.49	30.50 ± 0.47
Rosette diameter (cm)	21.30 ± 0.46	13.90 ± 0.55	14.20 ± 0.81	15.80 ± 1.04	20.30 ± 0.78	15.90 ± 0.55	15.30 ± 1.20	24.00 ± 0.40	16.40 ± 0.74	11.50 ± 0.45
Number of siliques	7.8 ± 1.6	916.2 ± 30.9	685.6 ± 21.9	425.4 ± 75.5	0.0 ± 0.0	352.0 ± 60.0	654.8 ± 102.9	0.0 ± 0.0	312.0 ± 19.6	618.8 ± 89.6
Average number of seeds per silique	0.0 ± 0.0	64.3 ± 1.4	62.9 ± 4.1	50.4 ± 4.2	0.0 ± 0.0	53.3 ± 2.5	58.3 ± 4.0	0.0 ± 0.0	63.0 ± 3.4	69.4 ± 4.3
Average number of seeds per plant	0 ± 0	58,808 ± 1605	43,209 ± 3447	22,989 ± 6219	0 ± 0	19,026 ± 3718	39,123 ± 7550	0 ± 0	19,765 ± 1774	44,834 ± 8583
**Below-ground trait measurements (*n* = 5)**										
Root length (cm)	21.06 ± 0.70	20.07 ± 1.86	12.53 ± 0.97	18.10 ± 1.10	18.62 ± 0.95	18.71 ± 0.55	17.45 ± 0.50	19.37 ± 0.73	19.26 ± 0.61	17.04 ± 0.41
Root surface area (cm^2^)	3.83 ± 0.07	3.02 ± 0.42	2.06 ± 0.41	2.95 ± 0.20	3.75 ± 0.08	3.60 ± 0.06	3.53 ± 0.09	3.70 ± 0.11	3.70 ± 0.09	3.70 ± 0.04
Root volume (cm^3^)	0.0554 ± 0.0026	0.0368 ± 0.0072	0.0290 ± 0.0085	0.0390 ± 0.0045	0.0608 ± 0.0024	0.0554 ± 0.0027	0.0568 ± 0.0024	0.0564 ± 0.0028	0.0568 ± 0.0021	0.0640 ± 0.0012
Average root diameter (mm)	0.58 ± 0.02	0.47 ± 0.04	0.50 ± 0.07	0.52 ± 0.03	0.65 ± 0.02	0.62 ± 0.02	0.65 ± 0.02	0.61 ± 0.02	0.61 ± 0.02	0.69 ± 0.01
**Training phase, monocultures (*n* = 5 pots with 10 plants each)**										
Above-ground biomass (g)	0.1302 ± 0.0086	0.1726 ± 0.0116	0.2138 ± 0.0132	0.1245 ± 0.0050	0.1128 ± 0.0181	0.0946 ± 0.0079	0.2162 ± 0.0077	0.0908 ± 0.0060	0.2276 ± 0.0075	0.1386 ± 0.0061
Stem height (cm)	3.60 ± 0.78	36.56 ± 1.53	39.15 ± 1.33	23.04 ± 1.04	2.19 ± 0.97	18.02 ± 2.90	45.70 ± 1.08	0.52 ± 0.35	27.80 ± 1.72	18.45 ± 0.30
Rosette diameter (cm)	11.15 ± 0.16	10.41 ± 0.27	9.30 ± 0.24	10.06 ± 0.39	11.84 ± 0.55	10.58 ± 0.10	8.45 ± 0.16	11.31 ± 0.32	11.54 ± 0.20	8.56 ± 0.43
Number of siliques	0.0 ± 0.0	63.3 ± 9.6	101.7 ± 9.0	19.5 ± 3.6	2.0 ± 1.3	10.1 ± 3.4	65.0 ± 2.9	0.3 ± 0.3	14.3 ± 2.2	63.6 ± 15.4
**Training phase, mixtures (*n* = 10)**										
Above-ground biomass (g)	0.3458 ± 0.0277	0.2513 ± 0.0286	0.2710 ± 0.0426	0.2021 ± 0.0128	0.1952 ± 0.0308	0.1481 ± 0.0335	0.2679 ± 0.0523	0.0849 ± 0.0102	0.5556 ± 0.0653	0.0488 ± 0.0128
Stem height (cm)	7.70 ± 1.94	39.30 ± 1.07	43.30 ± 1.34	27.45 ± 0.80	4.65 ± 3.18	22.00 ± 3.26	47.85 ± 1.22	0.00 ± 0.00	39.10 ± 1.03	17.30 ± 2.76
Rosette diameter (cm)	13.25 ± 0.65	10.73 ± 0.45	9.40 ± 0.31	11.70 ± 0.44	11.65 ± 0.65	10.45 ± 0.41	9.50 ± 0.66	11.65 ± 0.42	14.45 ± 0.64	7.20 ± 0.51
Number of siliques	0.2 ± 0.2	112.2 ± 10.5	119.8 ± 15.7	37.0 ± 4.6	7.1 ± 6.5	19.7 ± 8.6	87.4 ± 11.6	0.0 ± 0.0	46.7 ± 10.9	39.4 ± 8.1
**Experiment phase, monocultures on home soil (*n* = 3 pots with 10 plants each)**										
Above-ground biomass (g)	0.0561 ± 0.0049	0.1086 ± 0.0175	0.0834 ± 0.0166	0.0665 ± 0.0143	0.0451 ± 0.0099	0.0655 ± 0.0033	0.1840 ± 0.0413	0.0691 ± 0.0032	0.1107 ± 0.0215	0.0794 ± 0.0126
Stem height (cm)	6.90 ± 1.89	32.45 ± 1.15	36.38 ± 0.31	26.45 ± 1.93	6.60 ± 0.13	16.22 ± 2.48	45.14 ± 3.87	0.00 ± 0.00	35.13 ± 2.06	20.55 ± 0.57
Rosette diameter (cm)	6.42 ± 1.45	5.92 ± 0.52	5.03 ± 0.41	6.17 ± 1.33	7.05 ± 0.78	6.34 ± 0.21	6.25 ± 0.63	9.14 ± 0.43	9.28 ± 0.96	6.82 ± 0.39
Number of siliques	2.4 ± 1.2	90.8 ± 4.0	62.0 ± 2.1	57.1 ± 13.0	12.5 ± 3.1	19.0 ± 4.2	86.8 ± 21.1	0.0 ± 0.0	39.0 ± 8.1	112.1 ± 0.6
**Experiment phase, monocultures on away soil (*n* = 6 pots with 10 plants each)**										
Above-ground biomass (g)	0.0701 ± 0.0120	0.1271 ± 0.0206	0.1068 ± 0.0067	0.0694 ± 0.0064	0.0900 ± 0.0069	0.0919 ± 0.0138	0.0975 ± 0.0156	0.0695 ± 0.0200	0.1463 ± 0.0115	0.0796 ± 0.0050
Stem height (cm)	11.81 ± 0.90	33.70 ± 1.23	38.89 ± 1.00	23.77 ± 0.68	6.06 ± 1.25	24.09 ± 3.93	46.29 ± 0.55	0.00 ± 0.00	35.37 ± 1.38	19.63 ± 0.25
Rosette diameter (cm)	7.63 ± 0.41	6.47 ± 0.39	6.61 ± 0.45	5.96 ± 0.50	9.29 ± 0.31	6.87 ± 0.23	4.96 ± 0.38	9.19 ± 0.97	9.56 ± 0.67	5.96 ± 0.31
Number of siliques	3.7 ± 0.3	90.4 ± 9.7	90.1 ± 3.1	45.3 ± 3.9	18.0 ± 6.2	31.3 ± 8.3	59.5 ± 5.3	0.0 ± 0.0	51.7 ± 5.5	119.1 ± 3.4
**Experiment phase, mixtures (*n* = 5)**										
Above-ground biomass (g)	0.1194 ± 0.0390	0.0496 ± 0.0061	0.1175 ± 0.0278	0.0909 ± 0.0340	0.0331 ± 0.0070	0.0300 ± 0.0099	0.1470 ± 0.0131	0.0335 ± 0.0109	0.3003 ± 0.0830	0.0626 ± 0.0143
Stem height (cm)	13.90 ± 4.85	32.25 ± 0.76	37.90 ± 3.56	24.10 ± 2.46	0.00 ± 0.00	11.20 ± 4.18	40.40 ± 8.47	0.00 ± 0.00	42.90 ± 4.12	16.50 ± 1.51
Rosette diameter (cm)	7.40 ± 1.33	6.88 ± 0.37	6.10 ± 0.26	7.80 ± 0.90	7.20 ± 0.63	6.80 ± 0.94	5.10 ± 0.96	7.70 ± 0.81	10.10 ± 0.89	4.60 ± 0.48
Number of siliques	5.0 ± 3.0	70.3 ± 5.1	90.8 ± 18.8	32.6 ± 6.2	0.0 ± 0.0	4.6 ± 2.5	73.8 ± 17.1	0.0 ± 0.0	83.0 ± 21.6	84.0 ± 18.3

Regarding all measured above- and below-ground traits, the greatest dissimilarity emerged between Ct-1 and Te-0 (dissimilarity coefficient 0.786, see Table S2). On the other hand, Kin-0 and Tsu-0 were the most similar accessions (dissimilarity coefficient 0.112, see Table S2). However, to balance the design of the plant–soil feedback experiment, we did not always choose the most similar or dissimilar accession for the “away similar” and “away different” pairings, respectively. The mean dissimilarity coefficients of the final assignment were: 0.21 ± 0.02 for “away similar” and 0.43 ± 0.05 for “away different” (Table 2).

### Plant–soil feedback experiment

**Figure 3:**
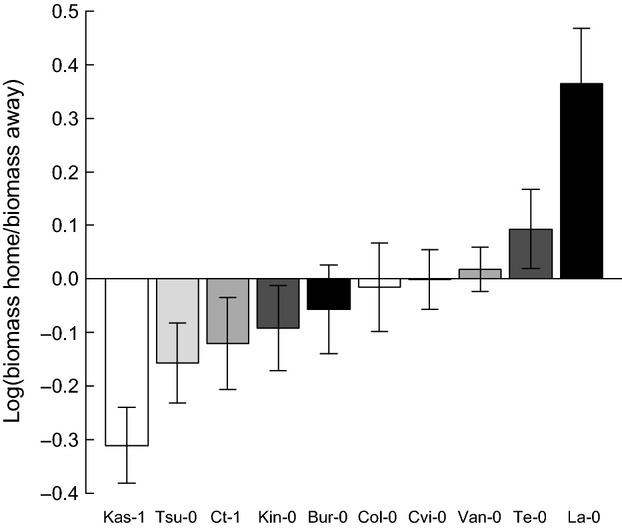
Average feedback experienced by the 10 *Arabidopsis thaliana* accessions. Negative values indicate negative feedback (smaller above-ground biomass in “home soil” than in “away soil”), positive values indicate positive feedback (higher above-ground biomass in “home soil” than in “away soil”). See “Statistical analyses” for detailed information on the calculation. Accessions are sorted by feedback strength, that is, from the strongest negative to the strongest positive feedback. Colors facilitate the comparison of accessions with Figure 2. However, please note that there is no statistically significant relationship between above-ground biomass and feedback strength (see Table S3 for the statistical analysis). Bars represent the mean ± standard error. *n* = 60 for each accession.

The *A. thaliana* accessions differed significantly in their feedback response (*F*_9,50_ = 5.004, *P* < 0.001, see Fig. 3 and Table S3). Four of the 10 accessions showed a negative feedback, that is, they had a higher biomass on “away soil” than on “home soil”: Kas-1, Tsu-0, Ct-1, and Kin-0 (Table 3). Among these accessions, Kas-1 had the strongest negative feedback. Four further accessions (Bur-0, Col-0, Cvi-0, and Van-0) were not affected by the soil type. On the other hand, two accessions showed positive feedback, that is, they had a smaller biomass on “away soil” than on “home soil”: La-0 and Te-0 (Table 3). We tested several indices (i.e., average above-ground biomass and average below-ground traits determined by the trait measurements, average above-ground biomass in monocultures and mixtures as well as relative above-ground biomass in mixtures determined during the training phase, latitude of the accessions' origins) that we expected to explain this variation between accessions. However, none of these indices explained the variance between accessions (Table S3). This can, for example, be seen by comparing Figures 2 and 3 in which differences in above-ground biomass (Fig. 2) and feedback strength (Fig. 3) do not show a consistent relationship.

**Figure 4:**
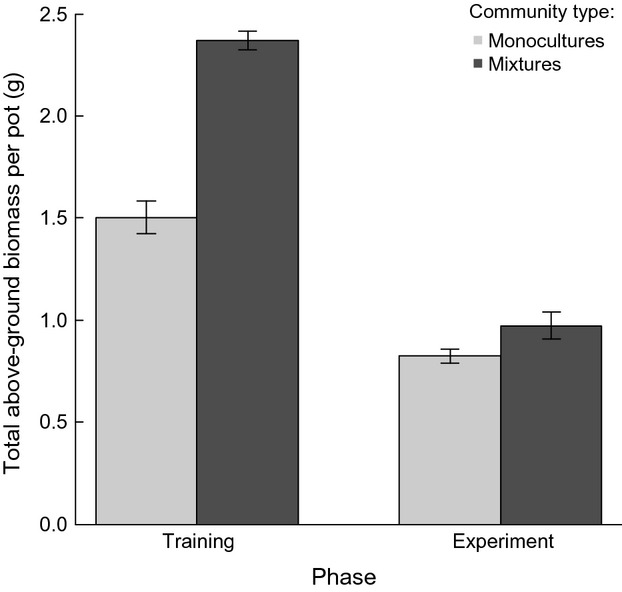
Effects of the phase, that is, training phase (two bars on the left) versus experiment phase (two bars on the right) as well as the community type, that is, monocultures (light gray) versus mixtures (dark gray), on the total above-ground biomass per pot in g. Bars represent the mean ± SE. *n*_monoculture training_ = 50, *n*_mixture training_ = 10, *n*_monoculture experiment_ = 90, *n*_mixture experiment_ = 5.

Figure 4 shows the results for total above-ground biomass per pot in the two phases (i.e., training phase vs. experiment phase) as well as community types (i.e., monocultures vs. mixtures). For both community types, total above-ground biomass per pot was significantly higher during the training phase than during the experiment phase (main effect “phase”: *F*_1,149_ = 154.110, *P* < 0.001; interaction “phase*community type”: *F*_1,149_ = 9.823, *P* = 0.002). Within each phase, the mixtures had a higher biomass than the monocultures (main effect “community type”: *F*_1,149_ = 30.384, *P* < 0.001). However, those differences were large and significant during the training phase, but small and nonsignificant during the experiment phase.

## Discussion

### Accession-specific trait variation

We found that the *A. thaliana* accessions differed significantly in the measured above- and below-ground traits. This result is in line with our hypothesis of strong intraspecific trait variation among the *A. thaliana* accessions and supports results from other studies (Mouchel et al. 2004; Passardi et al. 2007). We furthermore found differences between accessions in below-ground traits even though these were measured in seedlings instead of adult plants. As juvenile and adult plants typically show strong correlations of traits (Šmilauerová and Šmilauer 2007), we expect those differences to persist through subsequent life stages of the plants.

It seems reasonable to suppose that the differences between the accessions have a genetic basis. The expression of various genes might be the result of local adaptation to the respective climatic conditions of the accessions' origins leading to differences in root and shoot morphology (Weigel 2012). According to previous studies, *A. thaliana* accessions differ in their genome size (Schmuths et al. 2004) as well as in rather large gene regions (Clark et al. 2007; Ossowski et al. 2008), indicating correlations between genetic and morphological differences at intraspecific level. Whether intraspecific trait variation is smaller than interspecific trait variation is a controversial topic. Comparing various leaf traits, Roche et al. (2004), for example, found that interspecific variation is higher than intraspecific variation. Other reviews emphasize the importance of intraspecific diversity for ecological processes (Albert et al. 2011; Bolnick et al. 2011). In fact, an improved knowledge of the patterns of intraspecific and interspecific trait diversity is essential for a better understanding of intraspecific and interspecific interactions.

### Intraspecific plant–soil feedback

Many of the *A. thaliana* accessions in our experiment suffered from negative feedback, that is, the monocultures grew better on “away soil” than on “home soil.” Two accessions had positive feedback, with one (La-0) showing very strong positive feedback. These results support our hypothesis of plant–soil feedback at intraspecific level and differences in the strength of feedback experienced by the *A. thaliana* accessions. However, while those differences in feedback between the accessions were strong and significant, they could neither be explained by variation in the morphological traits nor by variation in the latitude of origin. Two crucial questions remain: What could have caused the feedback and why were there accession-specific differences in strength and direction of the feedback response?

In general, plant–soil feedback can be caused by biotic (microorganisms) or abiotic (allelochemicals, nutrients) factors (Ehrenfeld et al. 2005). For example, the different feedback responses could have been caused by soil microbes acting as plant mutualists or pathogens (Bever 2003; Bever et al. 2012; van der Putten et al. 2013). Recent studies have shown that *A. thaliana* accessions vary in the composition of their root microbiota (Bulgarelli et al. 2012; Lundberg et al. 2012). Thus, the negative feedback of some accessions found here might be due to a higher accumulation of accession-specific pathogens or a lower accumulation of accession-specific mutualists in “home soil” compared with the “away soils” and *vice versa* for positive feedback. Numerous studies have examined the soil biota of *A. thaliana* wild types and mutants in order to find specific genes that induce resistance to pathogens (Bisgrove et al. 1994; Aranzana et al. 2005). In general, *A. thaliana* is susceptible to infections by various groups of microorganisms such as viruses (Sosnová and Polák 1975), fungi (Koch and Slusarenko 1990a,b1990b), and bacteria (Simpson and Johnson 1990; Katagiri et al. 2002). Our plants did not show any visible damages on the shoots, and we do not know whether they were infested by below-ground herbivores or pathogens.

Generally, we view our methodology of using autoclaved soil without inoculum for the training phase as the most conservative approach. We would expect stronger effects with an experimental inoculum because of a larger number of soil organisms and species likely present in this inoculum. However, the decision what type of inoculum to use might greatly influence the results. For this reason, we decided to rely on natural colonization occurring in the greenhouse and surroundings being transported *via* air movements to colonize the soil and the plants to then affect the assembly of those soil communities, and did find feedback effects that developed during the training phase. The success of our approach without inoculum might also be related to the fact that *A. thaliana* is an early successional (pioneer) species and often colonizes new sites that might contain relative species-poor soil communities after disturbance.

Our intention was to reduce abiotic effects by mixing the trained soil types with nutrient-rich soil before the experiment phase. Despite this dilution, the possibility remains that the occurring plant–soil feedback was still at least partly caused by allelochemicals (van der Putten et al. 2013). Walker et al. (2003) identified various root exudates in the *A. thaliana* accession Col-0, and some of them were indeed allelopathic. As *A. thaliana* accessions are known to differ in their root exudates (Micallef et al. 2009), they possibly also differ in the exudation of allelopathic chemicals. Biedrzycki et al. (2010) found that individually growing *A. thaliana* seedlings respond differently when being exposed to root exudates of the same or another accession. In fact, seedlings growing in soil that contained the root exudates of another accession had longer primary roots and more lateral roots than those seedlings being exposed to root exudates of the same accession (Biedrzycki et al. 2010).

Furthermore, the plants might have suffered from nutrient deficiencies. This is supported by the fact that our plants had a smaller above-ground biomass during the experiment phase than during the training phase, similar to what was found by Aguilera et al. (2011). If the accessions differed in their nutrient requirements and consumption, plants growing on the trained soils might have suffered from nutrient-based abiotic negative feedback, which we cannot fully exclude based on our methodology. However, these effects should have been reduced strongly by adding 50% fresh (i.e., nutrient-rich) soil after the training phase.

Some accessions did not show any feedback response, and there were no differences in the plants' above-ground biomass between “away similar soil” and “away different soil.” There are several “core collections” of *A. thaliana* containing 8, 16, 24, 32, 40, or 48 available natural accessions being maximally genetically diverse among 265 selected accessions (McKhann et al. 2004). Half of the accessions chosen for our experiments (i.e., Bur-0, Ct-1, Cvi-0, Te-0, Tsu-0) belong to the “core collection 40,” that is, providing a certain a priori dissimilarity. However, several thousand natural accessions exist that might show larger variation in their morphological traits. Thus, the dissimilarity levels between accessions used here were potentially still too low to cause significant differences in performance between “home” and “away” (for some accessions) as well as between “away similar” and “away different” (for all accessions).

In recent years, substantial progress was made at describing and testing plant–soil feedback at species level. For example, we know that plant–soil feedback is more negative in native species compared with invasive species, in grassland species compared with tree species, in annuals compared with perennials, in early successional species compared with late successional species as well as in conspecifics compared with heterospecifics (Kulmatiski et al. 2008). Here, we show that intraspecific (interaccession) feedback operates, albeit possibly with smaller effect sizes and prevalence compared with interspecific feedback. Our experiment was using *A. thaliana* accessions as a model for demonstrating the existence of intraspecific soil feedback and examining its potential relationship with morphological traits. We do not suggest that these accessions actually coexist in nature. However, with climate change, anthropogenically influenced dispersal and related shifts of geographic ranges of species and populations, these encounters might occur in the future.

The next step could be to examine plant–soil feedback at various taxonomic levels of certain plant groups at the same time (from genotype to species to genus to family level). Conversely, trait differences instead of taxonomic differences could be the focus of feedback studies, however, potentially using larger trait differences for comparison than in our study.

### Intraspecific overyielding

Our hypothesis of intraspecific overyielding can broadly be confirmed. Overall, the mixtures had a higher above-ground biomass than the monocultures. However, in contrast to our expectation, the difference was significant during the training phase only, so feedback effects did not strengthen overyielding in our study. Despite the addition of nutrient-rich soil, nutrient conditions might have deteriorated from the training to the experiment phase. This could have reduced the possibilities for high-diversity mixtures to partition resources and overyield.

The diversity–productivity relationship has been shown to apply at the species level, that is, the higher the species diversity in a plant community, the higher the ecosystem productivity (Tilman et al. 1996, 2001; Loreau and Hector 2001; Hector et al. 2002; Kulmatiski et al. 2012; Hendriks et al. 2013). However, at intraspecific level, this relationship is controversially debated. For example, according to a study by Fridley and Grime (2009), the positive effects of a community consisting of plants belonging to different genotypes of one species were, if at all present, small compared with genotype monocultures. On the other hand, as shown by Reusch et al. (2005), intraspecific diversity might have positive effects on plant productivity. These different outcomes might be due to the fact that the species used in the two studies differed in their genetic diversity, that is, the genotypes examined by Reusch et al. (2005) could have been more genetically dissimilar than those examined by Fridley and Grime (2009). A more detailed accession–diversity–productivity experiment would be needed that uses gradual differences in accession diversity. Furthermore, mixture- and monoculture-trained soils could be used, and the study could specifically test whether observed diversity effects are weaker if monoculture-trained soil is sterilized before the experiment phase. This approach would help to quantify and disentangle the role of biotic, allelochemicals, and resources feedback effects in increasing ecosystem function. The outcome of a similar experiment performed at species level can indicate potential effect for the intraspecific case (Hendriks et al. 2013): The study used different diversity levels of soils and found that monocultures had a higher biomass when growing on mixed soil than on monoculture-trained soil. For the mixtures, differences were not significant, although.

## Conclusions

Our results provide some of the first evidence for intraspecific plant–soil feedback as well as a positive intraspecific diversity–productivity relationship. These findings demonstrate that intraspecific trait variation and intraspecific interactions may contribute to the maintenance of intraspecific diversity and therefore to community structure. In our experiment, negative feedback was slightly stronger and more prevalent than positive feedback, which corresponds to what is believed to apply at species level. Thus, although individual plants suffer from negative feedback, the plant community as a whole may benefit from it. In order to increase our understanding of this important connection, plant–soil feedback experiments should more often be combined with biodiversity experiments (Petermann et al. 2008; Hendriks et al. 2013). Plant–plant interactions may additionally be influenced by changes in climatic conditions as well as by below-ground herbivores and above-ground consumers (van der Putten et al. 2013), which may lead to variable feedback effects entailing multitude of possible consequences for the community. In conclusion, much complexity remains to be explored, especially when explicitly considering diversity and ecosystem functioning at intraspecific level.

## Supporting Information

**Table S1.** Detailed statistical results of the above- and below-ground trait measurements from MANOVA and ANOVAs. **Table S2.** Gower dissimilarity matrix of the 10 *Arabidopsis thaliana* accessions. **Table S3.** Detailed statistical results of the plant–soil feedback experiment from mixed-effects models.
