# Supplementary material for: Intraspecific plant–soil feedback and intraspecific overyielding in Arabidopsis thaliana
Source: Ecol Evol. 2014 May 24;4(12):2533–45. doi: 10.1002/ece3.1077 (PMC4203296; doi:10.1002/ece3.1077)
Supplement: Supplementary file 1 — Table S1. Detailed statistical results of the above- and below-ground trait measurements from MANOVA and ANOVAs. Table S2. Gower dissimilarity matrix of the 10 Arabidopsis thaliana accessions. Table S3. Detailed statistical results of the plant–soil feedback experiment from mixed-effects models. [file ece30004-2533-sd1.docx]

**Supporting Information**

**Table S1.** Detailed statistical results of the above- and below-ground trait measurements from multivariate analysis of variance (MANOVA) and univariate linear models (ANOVAs), respectively. Response variables are printed in bold. Mean values for “above-ground biomass” are visualized in Fig. 2. n=5 for each of the 10 *Arabidopsis thaliana* accessions. “Df”: degrees of freedom, “num Df”: numerator degrees of freedom, “den Df”: denominator degrees of freedom, “Sum Sq”: sums of squares, “Mean Sq”: mean squares

| **MANOVA: Above-ground biomass, stem height, rosette diameter, number of siliques, average number of seeds per silique, average number of seeds per plant, root length, root surface area, root volume, average root diameter** | | | | | |
| --- | --- | --- | --- | --- | --- |
|  | Df | approx. F | num Df | den Df | P value |
| Accession | 9 | 4.32 | 90 | 351 | <0.001 |
| Residuals | 40 |  | | | |
| **Above-ground biomass** | | | | | |
|  | Df | Sum Sq | Mean Sq | F value | P value |
| Accession | 9 | 5.984 | 0.665 | 11.253 | <0.001 |
| Residuals | 40 | 2.364 | 0.059 |  | |
| **Rosette diameter** | | | | | |
|  | Df | Sum Sq | Mean Sq | F value | P value |
| Accession | 9 | 658.92 | 73.21 | 21.206 | <0.001 |
| Residuals | 40 | 138.10 | 3.45 |  | |
| **Stem height** | | | | | |
|  | Df | Sum Sq | Mean Sq | F value | P value |
| Accession | 9 | 20782.6 | 2309.2 | 78.865 | <0.001 |
| Residuals | 40 | 1171.2 | 29.3 |  | |
| **Number of siliques** | | | | | |
|  | Df | Sum Sq | Mean Sq | F value | P value |
| Accession | 9 | 4726326 | 525147 | 28.278 | <0.001 |
| Residuals | 40 | 742832 | 18571 |  | |
| **Average number of seeds per silique** | | | | | |
|  | Df | Sum Sq | Mean Sq | F value | P value |
| Accession | 9 | 39431 | 4381.2 | 78.562 | <0.001 |
| Residuals | 40 | 2231 | 55.8 |  | |
| **Average number of seeds per plant** | | | | | |
|  | Df | Sum Sq | Mean Sq | F value | P value |
| Accession | 9 | 2.005∙e^10^ | 2227230164 | 17.748 | <0.001 |
| Residuals | 40 | 5.020∙e^9^ | 125489843 |  | |
| **Root length** | | | | | |
|  | Df | Sum Sq | Mean Sq | F value | P value |
| Accession | 9 | 243.43 | 27.05 | 5.013 | <0.001 |
| Residuals | 40 | 215.80 | 5.40 |  | |
| **Root surface area** | | | | | |
|  | Df | Sum Sq | Mean Sq | F value | P value |
| Accession | 9 | 13.890 | 1.543 | 5.763 | <0.001 |
| Residuals | 40 | 10.713 | 0.268 |  | |
| **Root volume** | | | | | |
|  | Df | Sum Sq | Mean Sq | F value | P value |
| Accession | 9 | 0.0061 | 0.0007 | 6.003 | <0.001 |
| Residuals | 40 | 0.0046 | 0.0001 |  | |
| **Average root diameter** | | | | | |
|  | Df | Sum Sq | Mean Sq | F value | P value |
| Accession | 9 | 0.224 | 0.025 | 4.247 | <0.001 |
| Residuals | 40 | 0.234 | 0.006 |  | |

**Table S2.** Dissimilarity matrix based on [Gower (1971](#_ENREF_22)) dissimilarity coefficients of the 10 *Arabidopsis thaliana* accessions. Coefficients were calculated with the R package “FD”, function gowdis() ([Laliberté & Shipley 2011](#_ENREF_33)). Higher coefficients mean higher dissimilarity. For reasons of clarity, in each row the highest coefficient is printed in bold and the smallest coefficient (different from zero) is printed in italics. The calculation used six above-ground traits (above-ground biomass, stem height, rosette diameter, number of siliques, average number of seeds per silique, average number of seeds per plant) and four below-ground traits (root length, root surface area, root volume, average root diameter). Based on these dissimilarity coefficients, we assigned each accession to one similar accession as well as to one dissimilar accession in the design of the plant-soil feedback experiment (Table 2)

|  | **Bur-0** | **Col-0** | **Ct-1** | **Cvi-0** | **Kas-1** | **Kin-0** | **La-0** | **Te-0** | **Tsu-0** | **Van-0** |
| --- | --- | --- | --- | --- | --- | --- | --- | --- | --- | --- |
| **Bur-0** | 0 | 0.591 | **0.705** | 0.450 | *0.121* | 0.302 | 0.441 | 0.142 | 0.350 | 0.515 |
| **Col-0** | 0.591 | 0 | 0.273 | 0.313 | 0.659 | 0.342 | *0.266* | **0.691** | 0.307 | 0.387 |
| **Ct-1** | 0.705 | *0.273* | 0 | 0.367 | 0.736 | 0.421 | 0.291 | **0.786** | 0.435 | 0.439 |
| **Cvi-0** | **0.450** | 0.313 | 0.367 | 0 | 0.448 | *0.183* | 0.277 | 0.419 | 0.292 | 0.291 |
| **Kas-1** | *0.121* | 0.659 | **0.736** | 0.448 | 0 | 0.317 | 0.459 | 0.136 | 0.389 | 0.478 |
| **Kin-0** | 0.302 | 0.342 | **0.421** | 0.183 | 0.317 | 0 | 0.153 | 0.365 | *0.112* | 0.278 |
| **La-0** | 0.441 | 0.266 | 0.291 | 0.277 | 0.459 | *0.153* | 0 | **0.512** | 0.193 | 0.222 |
| **Te-0** | 0.142 | 0.691 | **0.786** | 0.419 | *0.136* | 0.365 | 0.512 | 0 | 0.425 | 0.471 |
| **Tsu-0** | 0.350 | 0.307 | **0.435** | 0.292 | 0.389 | *0.112* | 0.193 | 0.425 | 0 | 0.335 |
| **Van-0** | **0.515** | 0.387 | 0.439 | 0.291 | 0.478 | 0.278 | *0.222* | 0.471 | 0.335 | 0 |

**Table S3.** Mixed effects analysis of plant-soil feedback (for details of the calculation of the feedback, see Material and Methods) testing a number of indices as explanatory variables (printed in bold) to explain the variance between accessions. Results for the explanatory variable “Accession (pot)” are shown in Fig. 3. Random effects are given in brackets. n=60 for each of the 10 *Arabidopsis thaliana* accessions. “num Df”: numerator degrees of freedom, “den Df”: denominator degrees of freedom

| **Accession (pot)** | | | | |
| --- | --- | --- | --- | --- |
|  | num Df | den Df | F value | P value |
| (Intercept) | 1 | 442 | 2.598 | 0.108 |
| Accession | 9 | 50 | 5.004 | <0.001 |
| **Above-ground biomass (accession)** | | | | |
|  | num Df | den Df | F value | P value |
| (Intercept) | 1 | 492 | 0.277 | 0.599 |
| Above-ground biomass | 1 | 8 | 0.490 | 0.504 |
| **Root length (accession)** | | | | |
|  | num Df | den Df | F value | P value |
| (Intercept) | 1 | 492 | 0.2582 | 0.612 |
| Root length | 1 | 8 | 0.0005 | 0.983 |
| **Root surface area (accession)** | | | | |
|  | num Df | den Df | F value | P value |
| (Intercept) | 1 | 492 | 0.259 | 0.611 |
| Root surface area | 1 | 8 | 0.017 | 0.898 |
| **Root volume (accession)** | | | | |
|  | num Df | den Df | F value | P value |
| (Intercept) | 1 | 492 | 0.259 | 0.611 |
| Root volume | 1 | 8 | 0.025 | 0.879 |
| **Average root diameter (accession)** | | | | |
|  | num Df | den Df | F value | P value |
| (Intercept) | 1 | 492 | 0.261 | 0.610 |
| Average root diameter | 1 | 8 | 0.099 | 0.762 |
| **Above-ground biomass in monocultures (accession)** | | | | |
|  | num Df | den Df | F value | P value |
| (Intercept) | 1 | 492 | 0.271 | 0.603 |
| Biomass in monocultures | 1 | 8 | 0.345 | 0.573 |
| **Above-ground biomass in mixtures (accession)** | | | | |
|  | num Df | den Df | F value | P value |
| (Intercept) | 1 | 492 | 0.272 | 0.602 |
| Biomass in mixtures | 1 | 8 | 0.379 | 0.555 |
| **Relative above-ground biomass in mixtures (accession)** | | | | |
|  | num Df | den Df | F value | P value |
| (Intercept) | 1 | 492 | 0.272 | 0.602 |
| Rel. biom. in mixtures | 1 | 8 | 0.373 | 0.558 |
| **Latitude (accession)** | | | | |
|  | num Df | den Df | F value | P value |
| (Intercept) | 1 | 492 | 0.340 | 0.560 |
| Latitude | 1 | 8 | 2.145 | 0.181 |
